# Toward a standardized multidisciplinary team approach in preterm infants at-risk for pulmonary hypertension

**DOI:** 10.1038/s41372-021-00949-3

**Published:** 2021-02-09

**Authors:** Anne C. Kesting, Georg Hansmann, Hannes Sallmon

**Affiliations:** 1 Department of Pediatric Cardiology, Charité—Universitätsmedizin Berlin, Berlin, Germany; 2 Department of Pediatric Cardiology and Critical Care, Hannover Medical School, Hannover, Germany; 3 Department of Congenital Heart Disease/Pediatric Cardiology, Deutsches Herzzentrum Berlin (DHZB), Berlin, Germany

**Keywords:** Paediatrics, Outcomes research

## To the Editor:

With great interest we read the recent article by Hébert et al. on practice variations in the management of pulmonary hypertension associated with bronchopulmonary dysplasia (BPD-PH) [1]. Despite improvements in neonatal care, BPD-PH remains a common condition with—clearly—higher mortality than BPD with normal pulmonary artery pressure [2].

Hébert et al. show that management of BPD-PH differs between N. American physicians of different subspecialties in several important aspects, such as timing of echocardiography and echocardiographic variables used to screen for BPD-PH [1]. While recent consensus statements recommend a multi-parametric echocardiographic approach to assess for the presence and severity of PH in infants with BPD, many physicians involved in neonatal care still rely on very few or even one “personal gold-standard” variable to assess right ventricular mass, function, and pressure [1–3]. Furthermore, the role of cardiac catheterization in advanced disease states needs to be further clarified. Invasive hemodynamics must be carefully weighed against potential risks, but may provide prognostic information (vasoreactivity, treatment response, detection of pulmonary vein stenosis) [2, 3]. The European Pediatric PVD Network recently developed a new algorithm for the management of BPD-PH, and recommends that diagnostic cardiac catheterization should be considered before (i) introduction of a second PH-specific medication and (ii) is advised if PH worsens under dual therapy or (iii) if unsatisfactory treatment response is seen in any infant with PH at a corrected postnatal age of >3 months [2, 3]. In some cases, additional lung function assessment may be required to adequately treat the underlying lung disease [2, 3].

Besides the diagnostic approach to BPD-PH, its optimal pharmacotherapy remains challenging. Currently, as shown by Hébert et al., the most commonly used first line medication is oral sildenafil [1], although it is not approved for infants <1 year of age by both FDA and EMA; thus, for BPD-PH, even oral sildenafil is administered off-label in most cases [2, 3]. The same holds true for endothelin receptor antagonists: Bosentan is approved for infants with pulmonary arterial hypertension above >1 year of age in Europe and >3 years of age in the U.S., but data on its safety and efficacy in young children with BPD-PH are sparse [2, 3]. Recently, we published the first pediatric prospective observational study on the use of macitentan, including five infants with BPD-PH [4]. We believe that further research on new PH-targeted therapies and the role of early combination therapies might help reduce morbidity and mortality in BPD-PH.

The profound practice variations among N. American physicians [1] illustrate that the key requirements for the establishment of internationally standardized diagnostic and therapeutic procedures have not yet been met. For example, the different definitions that are currently used for diagnosing BPD were shown to have significant impacts on morbidity and mortality, and likely preclude the comparability between studies [1, 2]. In addition, due to underpowered and retrospective studies, and the lack of randomized controlled trials, there is an urgent need for further high-quality research on BPD-PH.

To overcome some of these obstacles, we propose the establishment of multidisciplinary teams of neonatologists, cardiologists, pulmonologists, respiratory therapists, and allied health professionals that are involved early in the care of preterm infants at-risk for BPD-PH. These teams may facilitate adequate screening, treatment, and follow-up of PH in BPD infants in the first 2 years of life and beyond. A dedicated team approach has recently been shown to improve outcomes in severe BPD [5] and will likely also improve management and research efforts on BPD-PH.
